# Population health information research infrastructure—from data to public health actions

**DOI:** 10.1093/eurpub/ckae077

**Published:** 2024-07-01

**Authors:** Nienke Schutte, Petronille Bogaert, Miriam Saso, Herman Van Oyen

**Affiliations:** EU Health Information System Unit, Sciensano, Rue Juliette Wytsmanstraat 14, Brussels 1050, Belgium; EU Health Information System Unit, Sciensano, Rue Juliette Wytsmanstraat 14, Brussels 1050, Belgium; EU Health Information System Unit, Sciensano, Rue Juliette Wytsmanstraat 14, Brussels 1050, Belgium; EU Health Information System Unit, Sciensano, Rue Juliette Wytsmanstraat 14, Brussels 1050, Belgium

When the first COVID-19 cases were registered in Europe, the health information systems (HIS) of European countries were not ready to quickly adapt and respond to the crisis. Given the acuteness and novelty of this public health threat, data on what was effective against the virus were scarce. As a result, diverse policy responses and strategies were followed across the region causing the spread of contrasting messages and interventions, negatively impacting the health of the citizens. In times of crisis and beyond, the availability and trustworthiness of health information is indispensable.^1^ This edition, themed From Data to Public Health Actions, features work carried out by the Population Health Information Research Infrastructure (PHIRI).

The EU and its Member States need strong and resilient HIS that can deliver timely, sound and high-quality health information to support policymaking, strengthen (health) programme action and improve individual and population health outcomes.^2^^,^^3^ Both the European Commission and the Council of the European Union expressed the wish to examine how an improved alignment of health information activities at an EU level would function in terms of coherence, coordination and sustainability; data harmonization, collection, processing and reporting; research, capacity building; and transferability into evidence-based policy making.

The PHIRI provides powerful research tools and services to build collective intelligence, strengthen the public health workforce and build stronger HIS in order to increase preparedness and support the coordination of European efforts. This is achieved through a close collaboration with 41 partners across 30 countries. The PHIRI team is more than sum of its parts: it is a strong consortium that builds on almost a decade of collaboration and the achievements of BRIDGE Health^4^ and the Joint Action on Health Information (InfAct).^5^

PHIRI covers essential pillars to support strong and resilient HIS and research for evidence-based policy making across Europe. These are reflected in the papers covered in this special edition.

The first pillar focuses on the *exchange of health data, information and knowledge*. The European Health Information Portal (www.healthinformationportal.eu) serves as a one-stop shop facilitating the reuse through access to population health and health care data, information and expertise in Europe. The Portal includes metadata catalogues on health data sources, its providers and managers; trainings in population health information; European health information projects; and a dedicated COVID-19 policy measures corner. Furthermore, the PHIRI Rapid Exchange Forum allows for a quick exchange of knowledge and good practices during crises and to address urgent public health questions in peace time. Such a forum can be instrumental for upcoming work, e.g. defining indicators that need to be monitored for better resilience and preparedness. Finally, a sustainable, efficient HIS requires the development of new digital health information technologies managed by highly skilled professionals.

The second pillar focuses on *research and innovation*. PHIRI supports research across Europe through the identification, access, assessment and reuse of population health and non-health data to underpin public health policy decisions. A federated research infrastructure can overcome data reuse and data sharing hindrances for rapid policy-relevant research response. The current PHIRI Federated Research Infrastructure has been designed to comply with the General Data Protection Regulation, with each node being in charge of taking responsibility for compliance, minimizing the use of potentially sensitive data avoiding any personal data transmission in the federated analysis schema.

The third pillar focuses on *recommendations for policy actions*. When the COVID-19 crisis hit, the resilience of HIS differed substantially in European countries. PHIRI performed COVID-19 HIS assessments to learn important lessons from countries’ response to the pandemic and support the strengthening of HIS in Europe. In addition, public health foresight activities provided valuable information for decision makers to develop strategies and implement present day actions aiming at healthier futures. PHIRI developed a compact guide to support countries to develop their own public health foresight study. Such activities are a critical step to be better prepared for future crises and need to be performed on a regular basis.

In short, PHIRI is an integral research cornerstone for the implementation of the European Health Data Space (EHDS), aiming to invigorate the European health data ecosystem by facilitating tools and services for secondary use of health data. The three pillars presented in the papers are the services that the ‘PHIRI Research Infrastructure’ can provide to the research community to facilitate the reuse of data across countries, highlighting the large added value PHIRI can have for the EHDS. With the provision of its services, PHIRI will continue to build capacity across European countries, support ‘federated analysis of sensitive data’ and real-world data and consolidate ‘strong networks and platforms’ to collect information, start research projects and share expertise in an efficient manner.
